# Low-Intensity Ultrasound Causes Direct Excitation of Auditory Cortical Neurons

**DOI:** 10.1155/2021/8855055

**Published:** 2021-04-04

**Authors:** Xiaofei Qi, Kexin Lyu, Long Meng, Cuixian Li, Hongzheng Zhang, Lili Niu, Zhengrong Lin, Hairong Zheng, Jie Tang

**Affiliations:** ^1^Department of Physiology, School of Basic Medical Sciences, Southern Medical University, Guangzhou, China; ^2^Key Laboratory of Mental Health of the Ministry of Education, Southern Medical University, Guangzhou, China; ^3^Department of Anesthesiology, Shenzhen Maternity and Child Healthcare Hospital, Southern Medical University, Shenzhen, China; ^4^Guangdong-Hong Kong-Macao Greater Bay Area Center for Brain Science and Brain-Inspired Intelligence, Southern Medical University, Guangzhou, China; ^5^Department of Otolaryngology Head & Neck Surgery, Zhujiang Hospital, Southern Medical University, Guangzhou, China; ^6^Hearing Research Center, Southern Medical University, Guangzhou, China; ^7^Paul C. Lauterbur Research Center for Biomedical Imaging, Institute of Biomedical and Health Engineering, Shenzhen Institutes of Advanced Technology, Chinese Academy of Sciences, Shenzhen, China; ^8^Institute of Mental Health, Southern Medical University, Guangzhou, China

## Abstract

Cochlear implantation is the first-line treatment for severe and profound hearing loss in children and adults. However, deaf patients with cochlear malformations or with cochlear nerve deficiencies are ineligible for cochlear implants. Meanwhile, the limited spatial selectivity and high risk of invasive craniotomy restrict the wide application of auditory brainstem implants. A noninvasive alternative strategy for safe and effective neuronal stimulation is urgently needed to address this issue. Because of its advantage in neural modulation over electrical stimulation, low-intensity ultrasound (US) is considered a safe modality for eliciting neural activity in the central auditory system. Although the neural modulation ability of low-intensity US has been demonstrated in the human primary somatosensory cortex and primary visual cortex, whether low-intensity US can directly activate auditory cortical neurons is still a topic of debate. To clarify the direct effects on auditory neurons, in the present study, we employed low-intensity US to stimulate auditory cortical neurons in vitro. Our data show that both low-frequency (0.8 MHz) and high-frequency (>27 MHz) US stimulation can elicit the inward current and action potentials in cultured neurons. c-Fos staining results indicate that low-intensity US is efficient for stimulating most neurons. Our study suggests that low-intensity US can excite auditory cortical neurons directly, implying that US-induced neural modulation can be a potential approach for activating the auditory cortex of deaf patients.

## 1. Introduction

In mammals, the cochlear hair cells transduce the sound mechanical stimulation into electrical neural signals [1–3], which then be transferred by spiral ganglion neurons (SGNs) into the auditory cortex to have hearing ability. Previous studies have already shown that hair cells are very easy to be injured in response of various stresses, including ototoxic drugs, aging, noise, and inflammation [4–7]. The cochlear implant (CI) is a common treatment for hearing loss in children and adults, which can partially replace the function of hair cells. The multielectrode array converts acoustic signals into electrical signals which stimulate SGNs directly, activating auditory nervous system to generate hearing. This treatment requires anatomically intact cochlear nerves and normal function of SGNs for better outcomes [8]. Poor outcomes can be seen frequently in profound hearing loss patients with cochlear malformations or with cochlear nerve deficiencies due to the lack of accurate stimuli on SGNs by electrode. For example, deaf patients with neurofibromatosis type 2 (NF2), complete cochlear ossification, or cochlear nerve avulsion are not amenable to cochlear implant.

Auditory brainstem implant (ABI) or cranial nerve implants have been developed to restore auditory perception in these patients. The multielectrode array embedded within the brainstem stimulates the cochlear nucleus or higher stages of auditory nucleus directly, conferring the response of the central auditory system [9, 10]. However, because of the small number of electrodes and the broad region of neurons activated by each channel, the spatial selectivity of ABI is limited, restricting the outcomes of ABI [11]. Moreover, invasive surgery is required for ABI in the deep brain, increasing the complexity of surgery and the risk of complications [12]. Presently, only approximately 1,000 ABI procedures have been performed worldwide [11]. This number is far less than the population of deaf patients who are ineligible for CI. A noninvasive method stimulating the auditory nervous system is urgently needed.

Since the first observation of activation effects of ultrasound (US) on frog muscles in 1940, the effects of US stimulation on nerve system have been of great interests for neuroscientists [13, 14]. In the past decade, US has been demonstrated to modulate the neural activity in the thalamus, cortex, and hippocampus of different species, including humans [15–19]. Considering the penetrating and focusable characteristics of US stimulation, these findings suggest that US could be used as a noninvasive approach to modulating neural activity precisely [20, 21]. Based on these advantages, the concept of sonogenetics has been proposed as an alternative to optogenetics to advance the investigation and application of neuroscience [13, 14].

An obvious question that can be raised is whether US could replace electrical neural stimulation in ABI or cranial nerve implants. If central auditory neurons could be activated noninvasively by US, it is possible that the auditory response could be restored while avoiding the risk of craniotomy for many patients. Low-intensity US can result in the neural modulation in the human primary somatosensory cortex [22–24] and primary visual cortex [25], but whether US stimulation can directly activate the auditory cortex is not clear. In the present study, we examined the effects of low-intensity US stimulation on cultured auditory cortical neurons. Our data shows that low-intensity US is sufficient to elicit the excitation of single neurons. US stimulations with different frequencies are effective in activating most auditory cortical neurons. Our finding suggests that US is a potential approach to stimulating the auditory cortex safely and effectively, and further investigation of US stimulation as a method to restore hearing of patients suffering from hearing loss is meaningful.

## 2. Materials and Methods

### 2.1. Low-Intensity Ultrasound Stimulation

**Figure 1:**
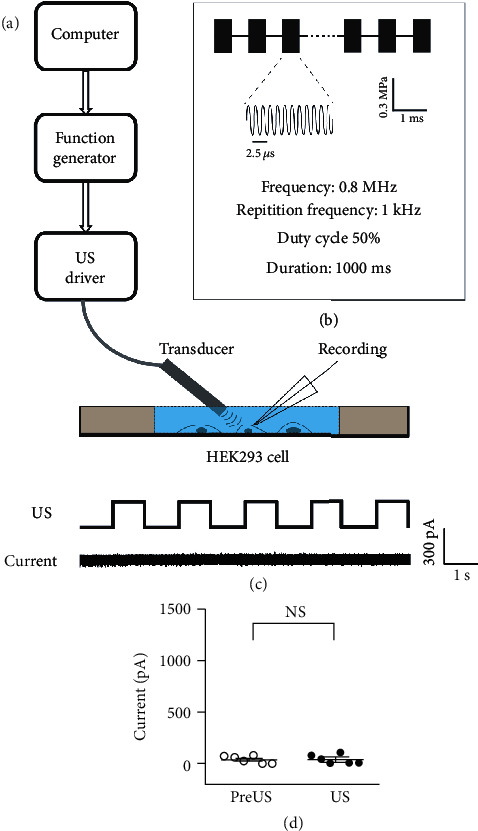
The ultrasound stimulation system and the patch clamp recording of cultured cells. (a) A schematic illustration of our combined recording and ultrasound system. The response to ultrasound stimulation of a single HEK293 cell was measured. (b) A schematic illustration of the pulsed waves of ultrasound stimulation, with an acoustic pressure of 0.3 MPa, 1 kHz repetition frequency, and 50% duty cycle. (c) US stimulation did not elicit changes in the membrane current of a representative HEK293 cell. (d) The mean current amplitude before and during US stimulation. Data are presented as the mean ± SE. NS: no statistic difference, *p* > 0.05, *n* = 7, paired *t*-test.

In the present study, a homemade ultrasound stimulation system was designed and used to stimulate single cultured cortical neurons or HEK293T cells. The pulsed ultrasound waves were generated by a computer-gated signal generator (RIGOL, DG4162) and amplified by a power amplifier (ZHL-5W-1, Mini-Circuits, Brooklyn, NY, USA). The ultrasound waves were then applied to the ultrasound transducer with a tip diameter of ~3 mm (Figure 1(a)). Each US stimulation contains 500 tone burst pulses at a center frequency of 0.8 MHz and a repetition frequency of 1 kHz with a duty cycle of 50% (Figure 1(b)). The interval between stimulus was 1 second. The peak-to-peak pressure was measured, and the output intensity was limited at 0.3 MPa. During the experiment, the transducer was tilted at 45° to the culture dish, and the tip of the transducer was submerged in the extracellular solution where the cells were located. Under a microscopy, the transducer was moved to the cell closely to stimulate the cell.

In some experiments, a custom-made ultrasound neuromodulation chip was used to generate surface acoustic waves. This chip consists of miniaturized interdigital transducers (IDTs) and an agar plate. The surface acoustic waves were generated from IDTs, and its wavelength was160 *μ*m at the resonant frequency of 27.42 MHz. The recording chamber where cells were located consisted of polydimethylsiloxane material with a diameter of 0.8 cm and a depth of 3 mm. The tone bursts of sinus ultrasound waves were generated by a computer-gated signal generator (RIGOL, DG4162), amplified by a power amplifier (ZHL-5 W-1, Mini-Circuits, Brooklyn, NY, USA) and applied to the IDTs. The ultrasound frequency, RPF, and voltage amplitude were controlled. The cells cultured on slips were placed in the chamber and received US stimulation for 1 s with a 9 s interval. The acoustic pressure generated by IDTs in the experiments was approximately 0.13 MPa measured by laser Doppler velocimetry (UHF-120 Ultra High-Frequency Vibrometer, Polytec, Germany).

### 2.2. Primary Cortical Neuron and HEK293T Cell Culture

Animal experiments were approved by the Animal Ethics Committee of Southern Medical University. For the primary culture of cortical neurons, fetal C57 mice were obtained at embryonic days 16-18. The whole brain was collected from fetal mice, and the auditory cortex was dissected and digested with 0.25% trypsin at 37°C for 10 min. The neurons were centrifuged and suspended in Dulbecco's modified Eagle's medium (DMEM, Gibco, Life Technologies, USA) with 10% FBS and plated at a density of 6 ~ 7 × 10^4^ cells/cm^2^ on poly-L-lysine (Sigma-Aldrich, St. Louis, MO, USA)-coated coverslips and cultured in a humidified 5% CO_2_ atmosphere at 37°C. After the neurons were adhered, the medium was changed to neurobasal medium (Gibco, life, USA) containing 2% B27 supplement (Gibco, life, USA). Afterwards, half of the medium was changed twice a week. At 14-18 days, the cells were removed for the experiments. HEK293T cells were cultured in DMEM (Gibco, Life Technologies, USA) supplemented with 10% fetal bovine serum (Gibco, Life Technologies, USA), as described previously [26, 27].

### 2.3. Electrophysiological Recording

The membrane current was recorded under whole-cell patch clamp recording mode as described in our previous study for different cell types [28–31]. In brief, under the whole-cell patch configuration, the membrane potential of neuron or HEK293T cell was held at -70 mV with a patch clamp amplifier (700B, Axon Instruments, USA). The membrane current before or during US stimulation was amplified, digitalized, and recorded by a patch clamp amplifier (700B) and a processor (1440A, Axon Instruments, USA). The extracellular solution contained (in mmol/L)150 NaCI, 1 MgCl_2_, 2.5 CaCl_2_, 10 HEPES, and 10 glucose, with a pH of 7.4 and osmotic pressure of 305 mmol/L. The intracellular solution of glass pipettes (resistance in the range of 2–5 M*Ω*) contained (in mmol/L) 140CsCl, 2MgCl_2_, 2 Mg-ATP, 1EGTA, 5HEPES, and 10 glucose with a pH of 7.35 and an osmotic pressure of 305 mmol/L.

The action potential of neurons was recorded under whole-cell patch clamp mode. After the configuration of the whole-cell patch, the cell was held at *I* = 0 under current clamp mode. The membrane potential before or during US stimulation was amplified, digitalized, and recorded by a patch clamp amplifier (700B) and a processor (1440A, Axon Instruments, USA). The extracellular solution contained (in mmol/L) 136 NaCl, 2.5 KCl, 1.3 MgSO_4_, 10 HEPES, 10 glucose, and 2.5 CaCl_2_ with a pH of 7.2-7.4 and an osmotic pressure of 290-310 mmol/L. The intracellular solution of glass pipettes (resistance in the range of 2–5 M*Ω*) contained (in mmol/L) KCl 130, Na-HEPES 10, EGTA 0.2, MgCl_2_ 2, with a pH 7.2, and osmotic pressure at 290-300 mmol/L.

For spike recording, the potential was recorded with the cell-attach (or loose patch) method using the same setup. When the tip of the recording electrode was attached to the membrane of the neurons, the neurons were held at *I* = 0 under the current clamp. The potential before or during US stimulation was amplified, digitalized, and recorded by a patch clamp amplifier (700B) and a processor (1440A, Axon Instruments, USA).

### 2.4. Immunocytochemical Fluorescent Staining

For MAP2 staining, after culturing for 14 days, the cortical neurons were fixed with 4% paraformaldehyde for 20-30 min at room temperature. After washing with PBS, the neurons were treated with 3‰ Triton X-100 for permeabilization. Then, the cells were blocked with 10% goat serum for 2 h. The neurons were then incubated with MAP2 primary antibody (1 : 1000, Proteintech, Chicago, IL, USA) in blocking buffer at 4°C overnight. After washing out the primary antibody with PBS, the neurons were incubated with a secondary antibody conjugated with Alexa Fluor568 (1 : 1000; goat-anti-rabbit, Life Tech, USA) in dark for 2 h at room temperature. Then, the cells were washed and mounted with ProLong Gold antifade mounting reagent (Invitrogen, Carlsbad, CA) on a glass slide. The fluorescence images were acquired using a confocal microscope (A1+, Nikon, Japan).

Detection of c-Fos expression was performed in neuron cultures with or without US stimulation. The neurons on the coverslip were fixed and treated with 3‰ Triton X-100. For neurons receiving US stimulation, this step should be performed within 30 min after stimulation. Then, the neurons were incubated with c-Fos primary antibody (BS1130, Bioworld Tech) at 4°C overnight. After washing out the primary antibody with PBS, the neurons were incubated with a secondary antibody conjugated with Alexa Fluor568 (1 : 1000; goat-anti-rabbit, Life Tech, USA) in dark for 2 h at room temperature. The fluorescence images were acquired, and the fluorescence intensity was calculated using a confocal microscope (A1+, Nikon, Japan).

### 2.5. Data Analysis and Statistics

The data analysis was performed using SPSS 22.0 (IBM, USA). A paired *t*-test was performed between the pre- and post-US stimulation groups; a two-sample *t*-test was performed between two groups. A repeated-measures ANOVA was applied when comparing two groups at different time points. Significance was defined as *p* < 0.05. GraphPad Prism 7 (GraphPad Software, San Diego, CA) was used for plotting.

## 3. Results

A customized ultrasound (US) stimulation system was used to stimulate the cultured cells. As shown in Figure 1(a), the US waves were delivered through a US transducer, which was submerged in the extracellular solution at a 45-degree angle to the bottom of the recording dish. This system results in a direct US stimulation to the recording cells and minimized acoustic reverberation. Each US stimulus comprised 500 tone burst pulses as shown in Figure 1(b). The center frequency of US stimulus was set at 800 kHz with a duty cycle of 50% at a repetition frequency of 1 kHz. The acoustic pressure was set at 0.3 MPa to minimize any possible thermal effects. By using a micromanipulator, the tip of the US transducer and the recording electrode were placed in the same view under the microscope such that the responses of the US-stimulated cell could be recorded by the patch-clamp recording system.

First, the possible effects of US stimulation were examined in the HEK293T cells. After the achieving whole-cell configuration, US stimulation was delivered to the recorded cell every other second for 20 s. Figure 1(c) shows a 10 s membrane current trail of a representative HEK293T cell. No detectable transmembrane current was found during the whole measurement during either US stimulation or the non-US period. Comparing the average membrane current during US stimulation (US, 38.33 ± 18.3 pA, mean ± SE) and the intervals of US stimuli (pre-US, 40.00 ± 14.4 pA, mean ± SE), no difference made by US was found for the seven HEK293T cells recorded (Figure 1(d), *p* = 0.96, the paired *t*-test). We thus confirmed that the low power US stimuli elicited no significant effect on HEK293T cells, including any possible changes to whole-cell patch configuration or thermal effects.

**Figure 2:**
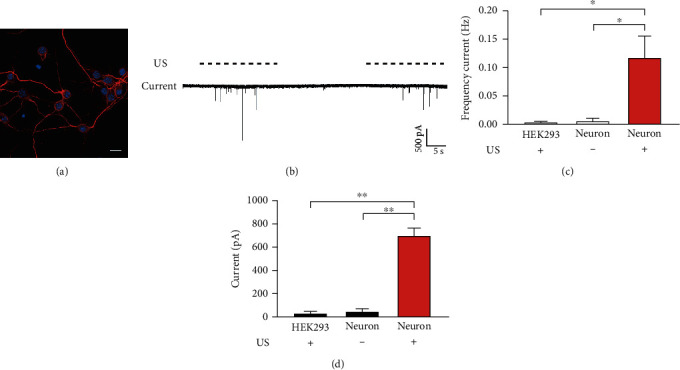
Ultrasound stimulation induces inward current in cultured cortical neurons. (a) A representative confocal image shows the immunofluorescence of MAP2 (red), a marker of mature neurons, of the cultured neurons after 14 days *in vitro*. The nuclei were labeled by DAPI (blue). Bar = 20 *μ*m. (b) The membrane current recording of a representative neuron in response to US stimuli. The dashed lines show the stimulation of US pulses. (c, d) The mean frequency (c) and amplitude (d) of inward current of neurons with or without US stimulation. The response of HEK293 cells is compared as the control. Data are presented as the mean ± SE. ^∗^*p* < 0.05; ^∗∗^*p* < 0.01; *n* = 6 for each group, one-way ANOVA.

The effects of US stimulation on auditory cortical neurons were examined in primary neuron cultures. Cultures of primary cortical neurons were prepared from the mouse auditory cortex on embryonic day (E) 17 [32, 33]. Dissected cells were cultured in neurobasal medium for at least 14 days to remove the neuroglial cells. We verified the composition of the cell culture by examining the immunofluorescence of MAP2, a marker of mature neurons. As shown in Figure 2(a), after culturing for 14 days in vitro (DIV), most cells were MAP2 positive, indicating that the culture was almost purely neural and the astrocytes and oligodendrocytes were negligible.

The responses of cultured neurons to US stimuli were examined by a whole-cell patch clamp at DIV 14 to 18.  Figure 2(b) shows the representative membrane current of a neuron in response to 0.3 MPa US stimuli. We observed no current change without US stimulation, whereas the neuron showed robust and large inward currents upon US stimuli. For six neurons measured, the mean frequency of the inward current (US+, 0.12 ± 0.04 Hz, mean ± SE) and their mean amplitude (US+, 694.5 ± 73.3 pA, mean ± SE) were significantly higher than those when US was absent (US-, *p* = 0.04 for frequency and *p* = 0.003 for amplitude, one-way ANOVA) (Figures 2(c) and 2(d)). Compared with the current changes recorded from HEK293T cells, the cortical neurons showed a significant response to US stimuli (*p* = 0.035 for frequency and *p* = 0.001 for amplitude, one-way ANOVA). Thus, these data confirmed that the cultured auditory cortical neurons could be activated by our US stimulation setup.

**Figure 3:**
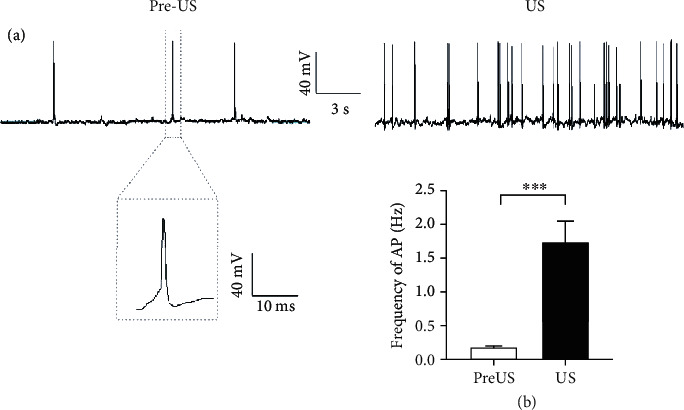
Ultrasound stimulation elicits action potentials in cultured cortical neurons. (a) Representative traces show the action potentials of a cultured cortical neuron before (pre-US) and during (US) US stimulation. Inset shows the shape of the action potential. (b) The mean frequencies of the action potentials of spontaneous firing (pre-US) and during (US) US stimulation. Data are presented as the mean ± SE. ^∗∗∗^*p* < 0.001, *n* = 9, paired *t*-test.

In the central auditory system, action potentials are critical for the information flow between neurons [34]. Therefore, we further measured the membrane potential to determine whether US stimulation could elicit action potentials of the culture neurons. Using the same US stimulation at the holding potential at -70 mV, we found that the representative neuron showed more action potentials during the period of delivered US stimulus (Figure 3(a)). Comparing with the spontaneous response, the number of action potentials for all nine neurons recorded increased significantly in response to US stimuli (Figure 3(b) 1.74 ± 0.33, mean ± SE, *p* < 0.001, paired *t*-test).

**Figure 4:**
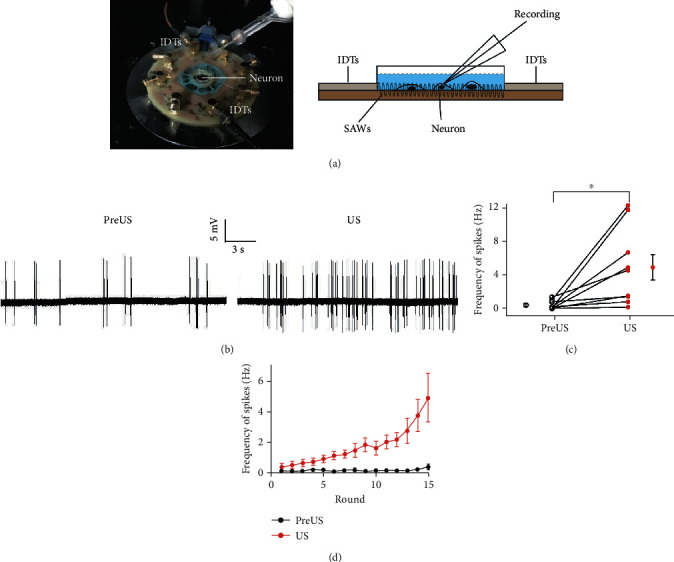
High-frequency ultrasound activates cultured auditory cortical neurons. (a) The ultrasound neural stimulation chip. Left, the photograph of the ultrasound neural stimulation chip used in the experiment. Right, a schematic illustration of the chip. The ultrasound neural stimulation chip consists of miniaturized interdigital transducers (IDTs) and an agar plate. The responses of neurons to surface acoustic waves (SAWs) were recorded. (b) The representative traces show the action potentials of a cultured cortical neuron before (pre-US) and during (US) US stimulation. (c) The changes of frequencies of action potential before (pre-US, open circles) and during (US, red circles) US stimulation for nine neurons. The mean values are also shown. Data are presented as the mean ± SE. ^∗^*p* < 0.05, *n* = 9, paired *t*-test. (d) The frequency of action potentials increased with the repeated US stimulation. Data are presented as the mean ± SE, *n* = 9.

Together with the inward current data, these results suggest that low-power and low-frequency US is sufficient to activate cortical neurons in vitro. Our finding is consistent with the reported results by measuring the low-frequency US-induced Ca^2+^ influx in brain slices [18]. However, several investigations have indicated that US stimuli with much higher frequency also produce remarkable biological effects on elegans and rat hippocampal neurons [35–37]. To determine whether high-frequency US can activate auditory cortical neurons as well, we employed the same ultrasound chip to deliver US to the neurons as described in previous research [35, 37]. This ultrasound chip generated surface acoustic waves such that the neurons attached on a region of the bottom of slice were stimulated by US with a resonant frequency of 27.42 MHz (Figure 4(a)). The spikes of stimulated neurons were recorded by the cell attached recording method, by which the long-term neural responses to US could be monitored.  Figure 4(b) shows the spikes of a representative neuron before and during US stimulation. Before US was delivered, the neuron showed some spontaneous spikes with a low firing rate (pre-US in Figure 4(b)). We found that its firing rate was increased by several rounds of US stimulation (US in Figure 4(b)). For all nine neurons examined, the mean firing rate after 15 rounds of US stimuli was 4.91 ± 1.54 Hz (mean ± SE), which was significantly higher than the spontaneous firing rate (0.39 ± 0.17 Hz, mean ± SE) before US stimulation (Figure 4(c), *p* = 0.015, the paired *t*-test). We also noticed that their firing rates were gradually increased with the rounds of US stimuli, implying the changes in excitation of the stimulated neurons (Figure 4(d)).

**Figure 5:**
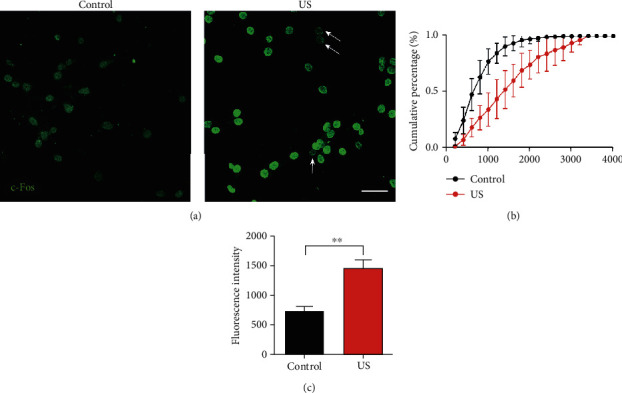
US stimulation increased c-Fos expression in auditory cortical neurons. (a) The representative confocal images show the immunofluorescence of c-Fos (green) of the cultured neurons with (right, US) or without (left, control) US stimulation. Arrows indicate the neurons without obvious increasing of c-Fos expression. Bar = 50 *μ*m. (b) Cumulative percentage of c-Fos fluorescence intensity with (red line) or without (black line) US stimulation. Data are presented as the mean ± SE, *n* = 5 cultures. (c) The mean intensity of c-Fos fluorescence of neurons with (red, US) or without (black, control) US stimulation. Data are presented as the mean ± SE. ^∗∗^*p* < 0.01, *n* = 5, Student's *t*-test.

To further determine the effects of US on the overall neurons, the c-Fos expression was examined for all neurons in the stimulation region on the slice. As an immediate early gene, c-Fos sensitizes to neural activity, resulting in the accumulation of c-Fos protein in the activated neurons [38]. As shown in the confocal images of c-Fos immunofluorescence (Figure 5(a)), after 5 min of US stimulation, the increased fluorescence of c-Fos was observed in most neurons with very few exceptions (white arrows in Figure 5(a)). We calculated the intensity of c-Fos immunofluorescence for all neurons. Compared with the controls without US stimulation, the curve of cumulative fluorescence intensity was shifted to the right by US (Figure 5(b), *p* < 0.001, two-way ANOVA). The mean fluorescence intensity was increased from 742.7 ± 81.1 (mean ± SE) to 1462.5 ± 147.7 (mean ± SE) after US stimulation, indicating that high-frequency US activated neurons significantly (Figure 5(c), *p* < 0.0027, Student's *t*-test).

## 4. Discussion

US stimulation provides a theoretical advantage over electrical stimulation for neuronal stimulation because of its noninvasive nature. In the past 20 years, scientists have found that low-intensity ultrasound can result in transient modulation of neural activity as a safe brain stimulation modality [13, 14]. In many mammalian species, in vivo and in vitro US stimulation have been demonstrated to modulate the activity of thalamic [15, 19], cortical [16, 17], and hippocampal [17, 18] circuits. There is also evidence that low-intensity US can result in the same neuromodulation in the human primary somatosensory cortex [22–24] and primary visual cortex [25]. In the present study, for the first time, we demonstrated that US stimulation modulates single-neuron discharge in the cultured neurons from mouse auditory cortex (Figures 2, 3, and 4). Both focused US (Figures 2 and 3) and surface US waves (Figures 4 and 5) are efficient at activating auditory cortical neurons. We also found that high-frequency US stimulation is as efficient as low-frequency US. These results consistent with the reported findings in different brain regions and in genetically modified neurons [16, 37, 39]. Although our results were observed in mice, we expect that low-intensity US can be applied to modulate the neural activity in human auditory cortex.

The cause of hearing loss is extremely heterogeneous and mainly caused by hair cell malfunction [40–42], and CI can partially have the hair cells function to compensate the hair cell loss. Thus, for children and adults suffering from severe and profound hearing loss, CI is the first-line treatment for hearing rehabilitation. Through a multielectrode array implanted in the cochlea, CI treatment stimulates the peripheral auditory system directly, conferring the restoration of hearing. However, the outcomes of CI rely on the normal anatomy and function of cochlear nerves [8]. This requirement excludes a population of patients with malfunction or malformation of cochlear nerves from CI candidates. ABI or cranial nerve implants have been developed for these deaf patients [9, 10]. By placing a multielectrode surface array within the brainstem, the cochlear nucleus or higher stages of auditory nucleus are directly stimulated by the ABI device. Similar with the situation for CI, the number of electrodes in an ABI device is usually small (21 electrodes in ABI541, the latest ABI device of Cochlear Corporation, Sydney, Australia). Because a broad region of neurons is activated by each channel, the poor spatial selectivity restricts the outcomes of ABI. This may explain the highly variable results of over 1,000 ABI procedures performed worldwide to date [11]. Meanwhile, implant migration and the risk of postoperative complications of craniotomy, including CSF leak, cerebellar contusion, meningitis, and hydrocephalus, also limit ABI surgery from becoming a wide spread procedure such as the CI [11, 12]. Considering the population of deaf patients with a nonfunctional and/or unimplantable cochlea, alternative strategies for safe and effective neuronal stimulation are urgently needed.

With the physical advantages, US can be focused across the human body and skull bone to deep-brain regions with millimeter spatial resolutions as a nonsurgical approach [20, 21]. US stimulation overcomes some limitations of other brain stimulation techniques. Compared with electric-based stimulations, US stimulation does not require the implant of electrodes while providing improved spatial selectivity versus transcranial electric stimulation [43] and transcranial magnetic stimulation [44]. US stimulation does not need genetic modification of neurons, which is required by optogenetic neural stimulation. Therefore, US stimulation offers an alternative strategy for patients who are ineligible for CI and ABI surgery.

For the central auditory system, it appears that the auditory cortex may be the appropriate region for receiving US stimulation. The functional structure of the auditory cortex offers many advantages for safe and region-specific US neuromodulation. Unlike the subcortical nuclei (i.e., cochlear nucleus, inferior colliculus, and auditory thalamus) [33, 45, 46], the auditory cortex is located on the surface of brain, which can be more easily and precisely stimulated by US stimulation. Meanwhile, the frequency presentation and other functional maps are arranged along the surface of the auditory cortex [32, 34, 47]. Through geometry of the transducers and phased arrays of ultrasound, it is instrumental in modulating the auditory cortex in a region-specific way with a high spatial resolution. Certainly, the spatial configuration and miniaturization of the US device should be modified to suit the application in the future.

However, several recent studies have questioned whether US stimulation can directly stimulate action potentials of cortical neurons. When ultrasound was focused on the mouse brain, Sato et al. found that the auditory startle reflex was elicited rather than the direct activation of motor circuits [48]. Guo et al. observed auditory and somatosensory cortical activity when ultrasound was applied to the brain, but these brain activations were abolished when the cochlear pathway was eliminated [49]. They postulated that the skull resonances caused by ultrasound radiation pressure result in the responses of the cochlear hair cells, leading to the activation of the whole auditory pathway including the auditory cortex. The activity of other nonauditory cortical areas could be elicited by the cross-modal projections from the auditory system. Their findings are a big challenge to the idea that US stimulation can be used as an alternative strategy for ABI. If US stimulation cannot activate auditory neurons directly, US is invalid for patients lacking normal cochlear functions. Our data indicate that low-intensity US stimulation can activate auditory cortical neurons directly regardless of the frequency and other parameters of US stimuli (Figures 3 and 4). Our finding is supported by a study demonstrating that transcranial focused ultrasound can evoke the same motor responses in deaf knockout mice as in normal hearing mice [50]. Therefore, we propose that US stimulation is efficient at activating the auditory cortex, and the application of US neural stimulation is worthy of further investigation for deaf patients.

## 5. Conclusions

Both low-frequency (0.8 MHz) and high-frequency (>27 MHz) ultrasound stimulation can activate auditory cortical neurons in vitro. Low-intensity US-induced neural stimulation is efficient for most cultured neurons. Our study suggests that low-intensity US can directly excite auditory cortical neurons.

## Data Availability

Data will be made available on request to corresponding author.
